# Supplementary material for: A framework for evaluating clinical artificial intelligence systems without ground-truth annotations
Source: Nat Commun. 2024 Feb 28;15:1808. doi: 10.1038/s41467-024-46000-9 (PMC10902352; doi:10.1038/s41467-024-46000-9)
Supplement: Supplementary file 1 — Supplementary Information [file 41467_2024_46000_MOESM1_ESM.pdf]

Supplementary Material

A framework for evaluating clinical artificial intelligence systems without ground-truth annotations

Dani Kiyasseh[1,*], Aaron Cohen[2,3], Chengsheng Jiang[2], and Nicholas Altieri[2]

[1]Cedars-Sinai Medical Center, Los Angeles, USA
[2]Flatiron Health, New York City, New York
[3]New York University School of Medicine, New York City, New York
[*]danikiy@hotmail.com

Supplementary Note 1 - Additional Results

SUDO is a reliable proxy for model performance on Multi-Domain Sentiment dataset

We implemented SUDO on the Multi-Domain Sentiment dataset. This allowed us to determine the effectiveness of SUDO on a dataset with a known distribution shift and which contains ground-truth labels.

We trained an NLP model to classify the sentiment of book reviews (source domain) and deployed it on reviews of electronic devices (target domain). Since the reviews from these two domains exhibit some differences (e.g., in content and vocabulary), they follow a different data distribution. In Supplementary Fig. 1a, we present the distribution of the probability values generated by the NLP model for data points from the negative and positive class, colour-coded in blue and green, respectively. Such colour-coding is only possible in this setting because of the presence of ground-truth labels, which we will not have in our target use-case and an assumption which we will discard in later sections.

We found that deciles exhibit class contamination, each containing data points from multiple classes. For example, for AI-based probabilities, $p \approx 0$, the majority of the data points belonged to the negative class (blue) yet there still existed some data points which belonged to the positive class (green).

We present the area under the receiver operating characteristic curve (AUC) achieved by a pair of classifiers (in the distinct pseudo-label settings) *per decile* when evaluated on a fixed held-out set (see Supplementary Fig. 1c). SUDO suggested that data points with $p \rightarrow 0$ are more likely to stem from the negative class than from the positive class (Supplementary Fig. 1c). This can be seen by $\uparrow$ AUC achieved by a classifier trained on the former (AUC ≈ 0.68) compared to one trained on the latter (AUC ≈ 0.50). The reverse argument can be made for data points with $p \rightarrow 1$. At this point, it is worthwhile to mention the apparent asymmetry when comparing both ends of the probability spectrum. Specifically, the performance of a classifier trained on data points with $p \approx 1$ and pseudo-labelled as positive is worse (AUC ≈ 0.60) than one trained on data points with $p \approx 0$ and pseudo-labelled as negative (AUC ≈ 0.68). This is despite the fact that these probability deciles exhibit relatively little contamination from the opposite class. One hypothesis for this is class overlap; namely, data points ($p \approx 1$) pseudo-labelled as positive (and which in fact are positive) might share some similarities and thus overlap with existing labelled data points from the negative class. Therefore, a classifier that attempts to distinguish between the two learns an unfavourable decision boundary, thus leading to the lower performance. Such an example underscores the potential limitations associated with strictly pseudo-labelling data points with a single class. Our pseudo-label discrepancy avoids such limitations by explicitly accounting for pseudo-labels from all possible classes (i.e., negative and positive).

We also present the per-decile pseudo-label discrepancy: the difference in performance between classifiers trained in two settings with different pseudo-label assignments (Supplementary Fig. 1e). We colour-code the bars based on the setting in which higher classifier performance was achieved. The main takeaway here is that the pseudo-label discrepancy confirms typical expectations about the approximate distribution of classes along the probability spectrum. Data points where $p \rightarrow 0$ are more likely to belong to the negative class than positive class. The opposite holds true where $p \rightarrow 1$. Note that the pseudo-label discrepancy, more broadly, does *not* necessitate the presence of ground-truth labels for data points in the wild (our target data set of interest). Its purpose, in this context, is to help better determine which subset of model predictions are unreliable. For example, based on Supplementary Fig. 1e, we can choose a cutoff value of $|D| = 0.05$ for the pseudo-label discrepancy, thereby identifying data points in the interval $0.30 < p < 0.80$ as unreliable: data points whose assigned labels are likely to be incorrect.

Having shown that pseudo-label discrepancy aligns with expectations, we also quantified its relationship with the accuracy of AI-based predictions. We define accuracy as is done in the machine learning calibration literature[?], reflecting the proportion of data points per decile which belong to the positive class. The intuition is that if pseudo-label discrepancy strongly correlates well with accuracy (see Supplementary Fig. 1g, $|\rho| = 0.98$), then it can be thought of as a reliable gauge of the distribution of classes (or equivalently class contamination) per decile. This, in turn, suggests that the pseudo-label discrepancy can be reliably deployed on data points without ground-truth labels.

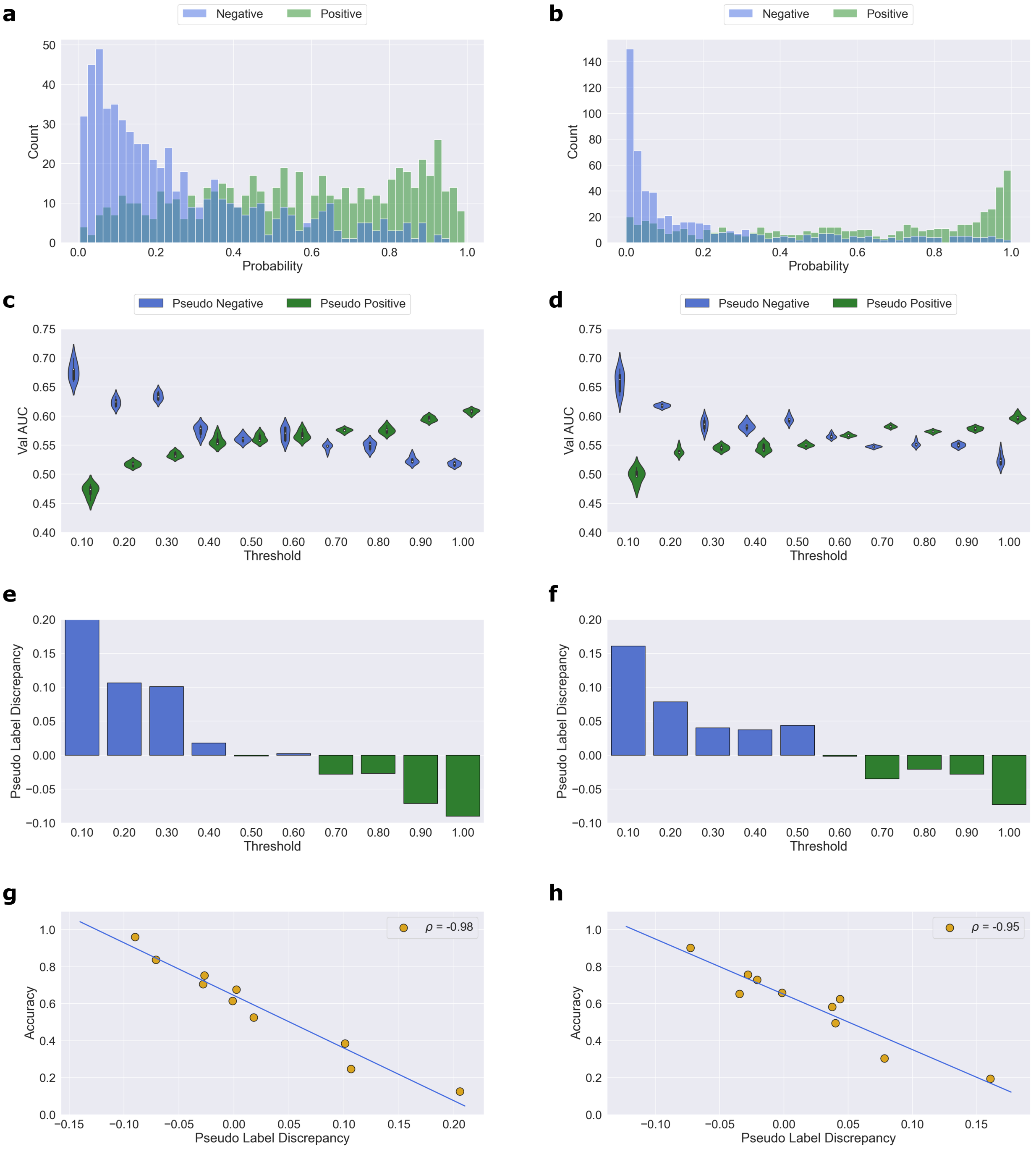

Supplementary Figure 1. SUDO produces a reliable proxy for model performance on Multi-Domain Sentiment dataset. An NLP model is trained to classify the sentiment of reviews about books (source domain) and deployed on reviews of electronics (target domain). Distribution of probability values output by an **(a)** ordinary and **(b)** overly-confident NLP model. **(c) - (d)** Performance of classifiers in the two pseudo-label discrepancy settings. **(e) - (f)** Pseudo-label discrepancy colour-coded based on the setting with higher performance. **(g) - (h)** Correlation, ρ, between pseudo-label discrepancy and proportion of data points in positive class per quantile. The high correlation indicates that the pseudo-label discrepancy metric is a reliable gauge of the distribution of classes (which we refer to as class contamination).

Exploring the limits of SUDO on simulated data

We implemented SUDO on simulated data in attempt to explore the limits of SUDO's applicability. We first experimented with multiple scenarios in which the data in the wild is varied by, for example, introducing an imbalance in the number of data points from each class (Supplementary Fig. 2b) or introducing data points from a third-and-unseen class (Supplementary Fig. 2c). The details of how we sampled the data can be found in the Methods section and the findings are summarized in the Results section.

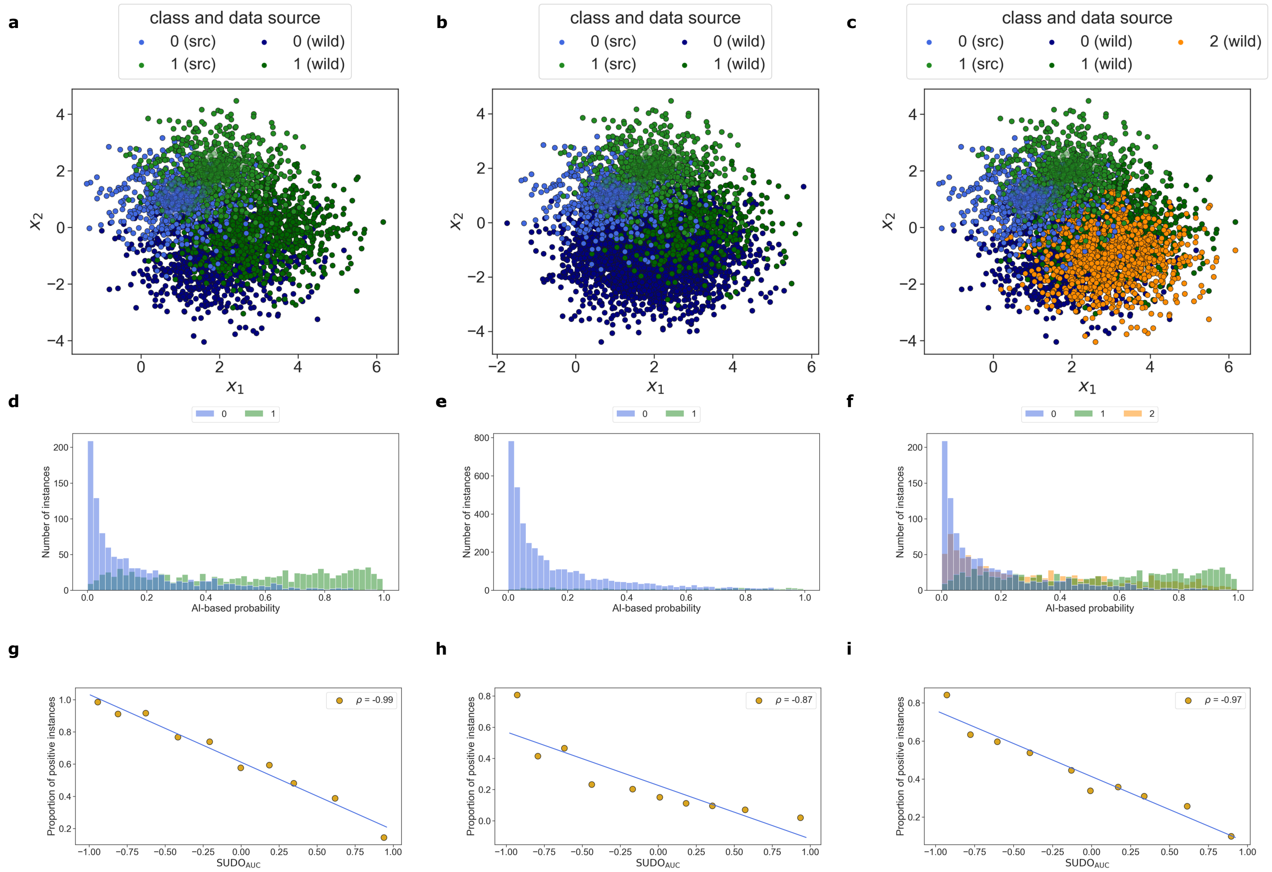

Supplementary Figure 2. SUDO continues to act as a reliable proxy for model performance under various scenarios. We implement SUDO on simulated data where the data in the wild exhibit (left column) distribution shift, (middle column) distribution shift with an imbalance in the number of data points from each class, and (right column) distribution shift with the presence of a third class. **(a-c)** Scatter plot of the data points in the training set (light shade) and in the wild (dark shade). **(d-f)** Distribution of the prediction probability values of models deployed on the data in the wild, colour-coded according to the ground-truth classes. **(g-i)** Correlation between the SUDO values and the proportion of positive instances in each of the probability intervals.

We conducted additional experiments to further probe the limits of SUDO's applicability. In particular, we explored the scenario in which the distribution of data points in the wild from one class remains fixed whereas the distribution of data points from the opposite class varied. In the baseline scenario (Supplementary Fig. 3a), we previously demonstrated that SUDO strongly correlates with the proportion of positive instances in each probability interval ($\rho = -0.99$). Note that the negative direction of this relationship is expected based on how we defined SUDO (see Supplementary Figs. 2g-i for additional evidence).

We then held fixed the distribution of the data points in the wild from class 0, and exclusively varied the mean of the distribution of the data points in the wild from class 1 (Supplementary Figs. 3b-c). The latter distribution was shifted to overlap with the former distribution (Supplementary Fig. 3b) and ultimately to swap its ordering (Supplementary Fig. 3c). We quantified this change as the distance of the mean from that in the baseline scenario (Supplementary Fig. 3a). We illustrate the correlation of SUDO with the proportion of positive instances as a function of these shifted distributions (Supplementary Fig. 3d). We found that SUDO no longer correlates ($\rho = 0$) with the proportion of positive instances when data points in the wild from opposite classes share similar features and are difficult to distinguish from one another. We also found that SUDO's relationship with the proportion of positive instances was inverted when the ordering of the distributions of data in the wild differs from that of training data. For example, $\rho \approx 0.65$ at a distribution change of $3\sqrt{2}$ (Supplementary Fig. 3d). Based on how we defined SUDO, this finding suggests that SUDO would erroneously indicate that the majority of data points in a probability interval belonged to class 0 when in reality they belonged to class 1. Although this scenario may be somewhat fictitious, it helps identify when SUDO should not be depended on.

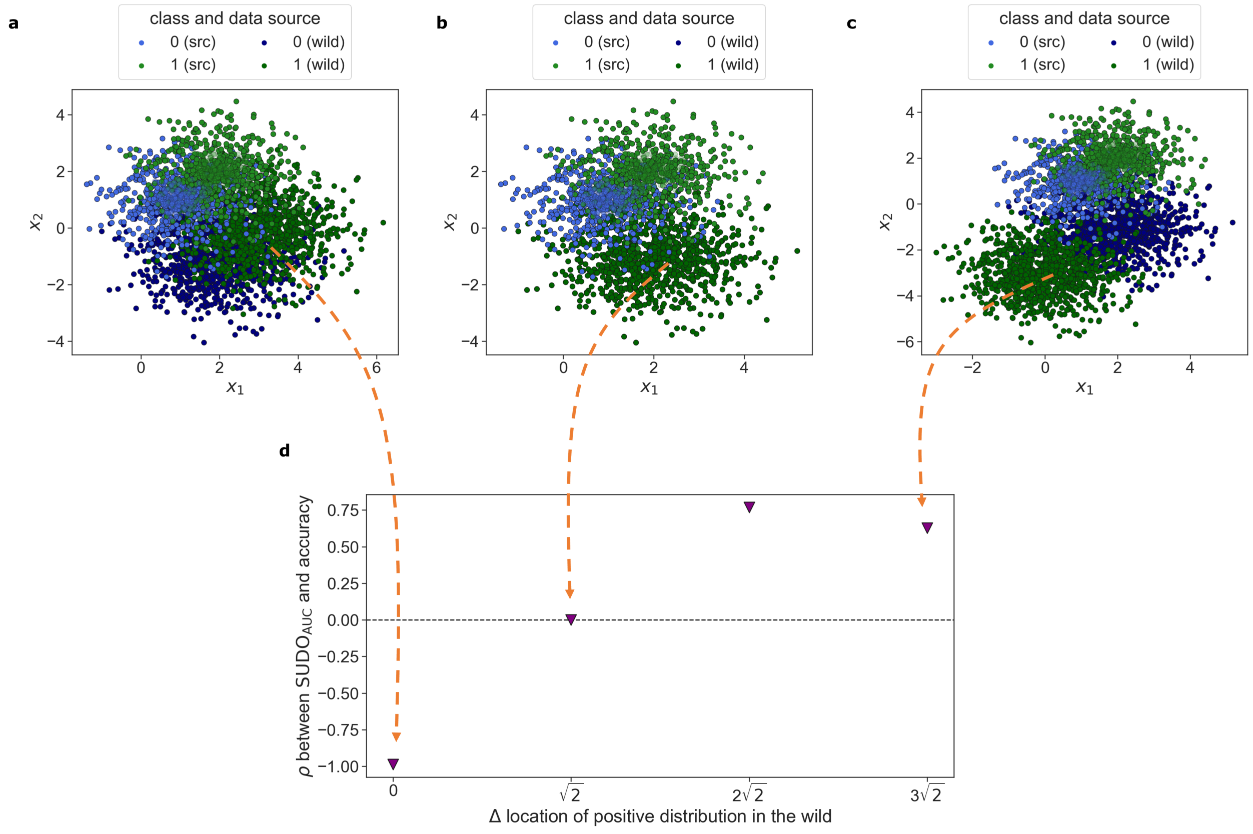

Supplementary Figure 3. SUDO can sometimes fail to act as a reliable proxy for model performance. We implement SUDO on simulated data where we hold fixed the distribution of data in the wild from class 0, and vary the distribution of data from the class 1. **(a-c)** Scatter plot of the data points in the training set (light shade) and in the wild (dark shade). **(d)** Correlation between the SUDO values and the proportion of positive instances as a function of the change in the mean of the distribution of data points from class 1. We show that extreme changes in that distribution can disrupt the reliability of SUDO as a proxy for model performance.

SUDO is insensitive to various hyperparameters

Here, we explore the sensitivity of SUDO to various hyperparameters. We implemented SUDO on the Flatiron Health ECOG PS dataset (without ground-truth annotations) having reduced the number of data points sampled from each probability interval (200 → 50, Supplementary Fig. 4b), and having changed the classifier used to distinguish between pseudo-labelled and ground-truth labelled data points (Fig. 4a - logistic regression vs. Fig. 4c - random forest). We show that these changes have a minimal effect on the correlation of SUDO with the proportion of positive instances in each probability interval.

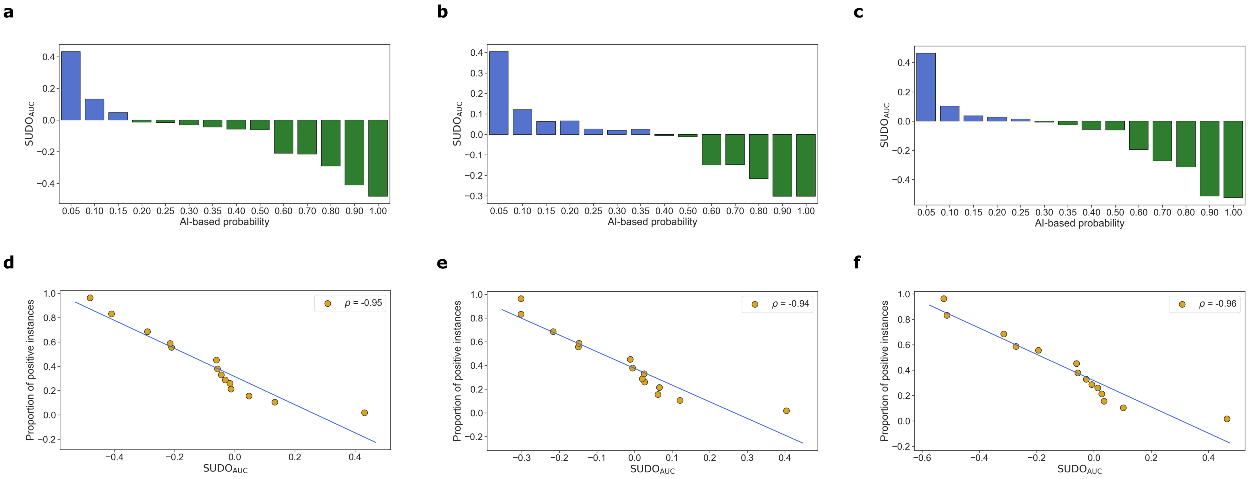

Supplementary Figure 4. SUDO is insensitive to various hyperparameters. (a-c) SUDO values on the Flatiron Health ECOG PS dataset with ground-truth annotations **(a)** having sampled 200 data points from each probability interval, **(b)** having sampled 50 data points from each probability interval, and **(c)** having used a different classifier to distinguish between pseudo-labelled and ground-truth labelled data points. **(d-e)** Correlation between the SUDO values and the proportion of the positive instances in each probability interval.

SUDO is agnostic to the metric used to evaluate classifiers

We claimed that SUDO works well with almost any metric used to evaluate the classifiers. Although we predominantly presented results for $SUDO_{AUC}$ in the main manuscript, we show that $SUDO_{Precision}$ and $SUDO_{ACC}$, where we used classifier precision and accuracy as the evaluation metrics, correlate equally well to the proportion of positive instances in each probability interval.

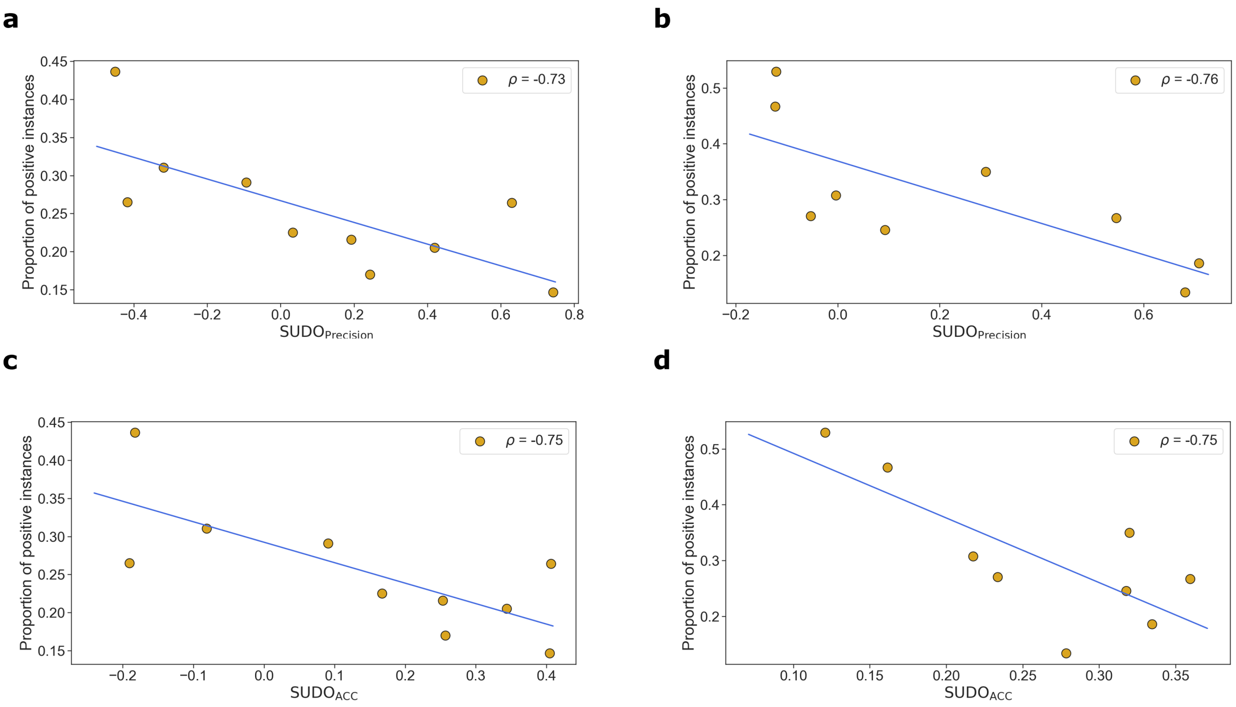

Supplementary Figure 5. SUDO is agnostic to the metric used to evaluate classifiers. Correlation between SUDO values and the proportion of positive instances in each probability interval on the Stanford DDI dataset. Results are shown for the (left column) DeepDerm and (right column) HAM10000 models. SUDO values are based on the **(a-b)** precision and the **(c-d)** accuracy of the classifiers.
