# Supplementary material for: A framework for evaluating clinical artificial intelligence systems without ground-truth annotations
Source: Nat Commun. 2024 Feb 28;15:1808. doi: 10.1038/s41467-024-46000-9 (PMC10902352; doi:10.1038/s41467-024-46000-9)
Supplement: Supplementary file 2 — Reporting Summary [file 41467_2024_46000_MOESM2_ESM.pdf]

nature portfolio

Corresponding author(s): Dani Kiyasseh

Last updated by author(s): Jan 26, 2024

Reporting Summary

Nature Portfolio wishes to improve the reproducibility of the work that we publish. This form provides structure for consistency and transparency in reporting. For further information on Nature Portfolio policies, see our Editorial Policies and the Editorial Policy Checklist.

Statistics

For all statistical analyses, confirm that the following items are present in the figure legend, table legend, main text, or Methods section.

n/a	Confirmed	
☐	☒	The exact sample size (*n*) for each experimental group/condition, given as a discrete number and unit of measurement
☐	☒	A statement on whether measurements were taken from distinct samples or whether the same sample was measured repeatedly
☐	☒	The statistical test(s) used AND whether they are one- or two-sided<br>*Only common tests should be described solely by name; describe more complex techniques in the Methods section.*
☐	☒	A description of all covariates tested
☒	☐	A description of any assumptions or corrections, such as tests of normality and adjustment for multiple comparisons
☐	☒	A full description of the statistical parameters including central tendency (e.g. means) or other basic estimates (e.g. regression coefficient) AND variation (e.g. standard deviation) or associated estimates of uncertainty (e.g. confidence intervals)
☐	☒	For null hypothesis testing, the test statistic (e.g. *F*, *t*, *r*) with confidence intervals, effect sizes, degrees of freedom and *P* value noted<br>*Give P values as exact values whenever suitable.*
☒	☐	For Bayesian analysis, information on the choice of priors and Markov chain Monte Carlo settings
☒	☐	For hierarchical and complex designs, identification of the appropriate level for tests and full reporting of outcomes
☒	☐	Estimates of effect sizes (e.g. Cohen's *d*, Pearson's *r*), indicating how they were calculated

Our web collection on statistics for biologists contains articles on many of the points above.

Software and code

Policy information about availability of computer code

Data collection	No software was used to collect data
Data analysis	The code used to conduct the experiments is available at https://github.com/flatironhealth/SUDO<br><br>The following were used for the analysis:<br>Python 3.0<br>PyTorch 1.8.0<br>Pandas 2.1.0<br>Numpy 1.23.0<br>Lifelines 2.8.0

For manuscripts utilizing custom algorithms or software that are central to the research but not yet described in published literature, software must be made available to editors and reviewers. We strongly encourage code deposition in a community repository (e.g. GitHub). See the Nature Portfolio guidelines for submitting code & software for further information.

Data

Policy information about availability of data

All manuscripts must include a data availability statement. This statement should provide the following information, where applicable:

- Accession codes, unique identifiers, or web links for publicly available datasets
- A description of any restrictions on data availability
- For clinical datasets or third party data, please ensure that the statement adheres to our policy

The Stanford diverse dermatology images (DDI) dataset is publicly-available at: https://ddi-dataset.github.io/index.html#access.
The Multi-Domain Sentiment dataset is publicly available at: https://www.cs.jhu.eduhndredze/datasets/sentiment/index2.html.
The Camelyon17-WILDS dataset is publicly available at: https://wilds.stanford.edu/get_started/
All publicly-available datasets were used as permitted, exclusively for research purposes.
The Flatiron Health ECOG PS dataset is available under restricted access due to patient privacy. Requests for data sharing by license or by permission for the specific purpose of replicating results in this manuscript can be submitted to PublicationsDataAccess@flatiron.com.
The data for generating the figures in this study are in the Source Data file.

Research involving human participants, their data, or biological material

Policy information about studies with human participants or human data. See also policy information about sex, gender (identity/presentation), and sexual orientation and race, ethnicity and racism.

Reporting on sex and gender	We describe the Flatiron Health ECOG PS dataset comprehensively in the Methods section to reflect its constituent patient population. The Institutional Review Board of WCG IRB (reference number: IRB00000533) approved of the study protocol prior to study conduct, and included a waiver of informed consent.
Reporting on race, ethnicity, or other socially relevant groupings	See above for Flatiron Health ECOG PS dataset.<br>For the Stanford DDI dataset, we used the original authors' definition of skin tone based on the Fitzpatrick Scale.
Population characteristics	See above for Flatiron Health ECOG PS dataset.
Recruitment	n/a
Ethics oversight	The Institutional Review Board of WCG IRB (reference number: IRB00000533) approved of the study protocol prior to study conduct, and included a waiver of informed consent.

Note that full information on the approval of the study protocol must also be provided in the manuscript.

Field-specific reporting

Please select the one below that is the best fit for your research. If you are not sure, read the appropriate sections before making your selection.

☒ Life sciences ☐ Behavioural & social sciences ☐ Ecological, evolutionary & environmental sciences

For a reference copy of the document with all sections, see nature.com/documents/nr-reporting-summary-flat.pdf

Life sciences study design

All studies must disclose on these points even when the disclosure is negative.

Sample size	For the following datasets, no calculations were made to determine sample size, and we used the samples provided by the original authors. For the Stanford DDI dataset, there are n=656 samples in total. For the Multi-Domain Sentiment dataset, there are n=2,000 samples from each domain (n=1,000 positive, n=1,000 negative). For the Camelyon17-WILDS dataset, there are n=450,000 samples in total.<br><br>For the Flatiron Health ECOG PS dataset, there are n=117,529 samples with ECOG PS labels and n=33,618 samples without ECOG PS labels. We chose this sample size based on the availability of clinical reports and ECOG PS labels.
Data exclusions	For the Stanford DDI dataset, we test on the entire dataset. For the Multi-Domain Sentiment dataset, we only focus on product reviews from the domains of 1) books and 2) electronics. For the Flatiron Health ECOG PS dataset, we provide details of inclusion and exclusion criteria in the Methods section of the manuscript.
Replication	To verify the repeatability and reproducibility of the experiments, we conducted them using five pseudo-random seeds (0, 1, 2, 3, 4, 5) using Python's random.seed() function. This ensured that the same experimental settings are repeated throughout.
Randomization	When selecting samples from each probability interval during the SUDO experiments, we used five pseudo-random seeds (0, 1, 2, 3, 4, 5) using Python's random.seed() function. This ensured we sampled the same set of samples for training and evaluating the classification models across the experiments.

Blinding	As we conducted a retrospective analysis of de-identified data, no blinding was necessary in this study.

Reporting for specific materials, systems and methods

We require information from authors about some types of materials, experimental systems and methods used in many studies. Here, indicate whether each material, system or method listed is relevant to your study. If you are not sure if a list item applies to your research, read the appropriate section before selecting a response.

Materials & experimental systems

n/a	Involved in the study	
☒	☐	Antibodies
☒	☐	Eukaryotic cell lines
☒	☐	Palaeontology and archaeology
☒	☐	Animals and other organisms
☒	☐	Clinical data
☒	☐	Dual use research of concern
☒	☐	Plants

Methods

n/a	Involved in the study	
☒	☐	ChIP-seq
☒	☐	Flow cytometry
☒	☐	MRI-based neuroimaging

Plants

Seed stocks	*Report on the source of all seed stocks or other plant material used. If applicable, state the seed stock centre and catalogue number. If plant specimens were collected from the field, describe the collection location, date and sampling procedures.*
Novel plant genotypes	*Describe the methods by which all novel plant genotypes were produced. This includes those generated by transgenic approaches, gene editing, chemical/radiation-based mutagenesis and hybridization. For transgenic lines, describe the transformation method, the number of independent lines analyzed and the generation upon which experiments were performed. For gene-edited lines, describe the editor used, the endogenous sequence targeted for editing, the targeting guide RNA sequence (if applicable) and how the editor was applied.*
Authentication	*Describe any authentication procedures for each seed stock used or novel genotype generated. Describe any experiments used to assess the effect of a mutation and, where applicable, how potential secondary effects (e.g. second site T-DNA insertions, mosiacism, off-target gene editing) were examined.*
